# Editorial: Networks in Movement Disorders. To Move or Not to Move

**DOI:** 10.3389/fneur.2021.758246

**Published:** 2021-10-14

**Authors:** Holm Graessner, Alberto Albanese, Ludger Schöls

**Affiliations:** ^1^Institute for Medical Genetics and Applied Genomics, University of Tübingen, Tübingen, Germany; ^2^Centre for Rare Diseases, University Hospital Tübingen, Tübingen, Germany; ^3^Department of Neurology, IRCCS Humanitas Research Hospital, Milan, Italy; ^4^Department of Neurodegenerative Diseases, Hertie Institute for Clinical Brain Research and Center of Neurology, University of Tübingen, Tübingen, Germany

**Keywords:** networks, movement disorders, rare movement disorders, care, research

Movement disorders are a growing field in clinical neuroscience with a strong translational value. Expert assessment is required for managing patients with movement disorders, which is more and more often being provided by multidisciplinary teams at expert centers due to the complexity of the diseases with regards to clinical features, diagnostics, and sophisticated tools to assess the course of disease and measure disease progression. There is need for scientific interaction among a variety of specialized domains and for patient-centered management of this quickly evolving area of knowledge.

Research-driven knowledge is constantly providing new information that directly influences clinical practice in the field of movement disorders. An increasing number of movement disorders will soon be treated by candidate therapeutics or devices ready for testing in clinical trials; thus, trial readiness for well-defined cohorts has become a highly important challenge. Lumping together patients with specific movement disorders subtypes may be necessary to reach sufficient power in trials and to implement best clinical practice.

Thus, collaborative networks have been formed to enable sharing of knowledge and data, further the development of clinical standards and of best practice, and enable pursuing research projects, particularly to develop innovative therapeutic approaches. It is a logical consequence that these networks focus on specific diseases or disease groups that allow the efficient and effective bringing together of key stakeholders (Table 1). A third logical consequence is that these collaborative networks are most frequent and successful in rare movement disorders for which the complexity and fragmentation inherent in health care and research is largest. Nine of the eleven manuscripts of this special issue actually focus on rare movement disorders. Networks in rare movement disorders, motivated by the need to put together patients and experts, very often have an international breadth.

**Table 1:** Overview of disease group focus of the *Networks in Movement Disorders* manuscripts.

**Manuscript**	**Disease group**	**Title of manuscript**
Reinhard et al.	Rare neurological diseases	The European Reference Network for Rare Neurological Diseases
Van de Warrenburg et al.	Rare movement disorders	The Architecture of Contemporary Care Networks for Rare Movement Disorders: Leveraging the ParkinsonNet Experience
Albanese et al.	Parkinson's disease	Design and Operation of the Lombardy Parkinson's Disease Network
Smit et al.	Dystonias	Dystonia Management: What to Expect From the Future? The Perspectives of Patients and Clinicians Within DystoniaNet Europe
Kilic-Berkmen et al.	Dystonias	The Dystonia Coalition: A Multicenter Network for Clinical and Translational Studies
Lin et al.	Spinocerebellar Ataxias (SCA)	Collaborative Efforts for Spinocerebellar Ataxia Research in the United States: CRC- SCA and READISCA
Traschütz et al.	Autosomal Recessive Cerebellar Ataxias (ARCA)	The ARCA Registry: A Collaborative Global Platform for Advancing Trial Readiness in Autosomal Recessive Cerebellar Ataxias
Kleimaker et al.	Tourette Syndrome	Networks in the Field of Tourette Syndrome
Karin et al.	Neurodegeneration with Brain Iron Accumulation (NBIA)	Treat Iron-Related Childhood-Onset Neurodegeneration (TIRCON)—An International Network on Care and Research for Patients With Neurodegeneration With Brain Iron Accumulation (NBIA)
Sathe et al.	Huntington's disease (HD)	Enroll-HD: an integrated clinical research platform and worldwide observational study for Huntington's disease
Respondek and Höglinger	Progressive supranuclear palsy (PSP)	DescribePSP and ProPSP: German Multicenter Networks for Standardized Prospective Collection of Clinical Data, Imaging Data, and Biomaterials of Patients With Progressive Supranuclear Palsy

Although collaborative networks are a very efficient method to address the issues mentioned above, the established networks do not implement a unique or similar conceptual and constitutional models. The experience and expertise, as well as exemplary concepts and solutions provided by the existing disease networks in movement disorders, promote mutual learning and broker expert knowledge for additional novel networks.

Despite differences in the networks and manuscripts, a few cross-cutting generic topics can be identified that are characteristic of collaborative networks in movement disorders and, moreover, determine the focal point of some of the manuscripts. Firstly, the issue of care organization, improvement, and operation is being discussed from different perspectives and for different disease groups in Van de Warrenburg et al., Reinhard et al., Smit et al., and Albanese et al..

Secondly, a multi-stakeholder oriented establishment of network and infrastructure to enable care improvement and research in a specific disease field is featured in Kleimaker et al. and Karin et al. Thirdly, the value of clinical research platforms for structuring research as well as achieving trial readiness is described in Sathe et al. and Traschütz et al. Finally, Lin et al., Respondek and Höglinger, and Kilic-Berkmen et al. focus on clinical and translational research that is done in collaborative networks.

Research networks represent a fundamental shift in the geography of science, as they not only facilitate integration of research in the era of globalization, but also provide a powerful tool for stimulating societal changes (1). The latter goal requires significant interactions between academic science and policy makers. Networks do not succeed naturally. Key enabling factors for the development of collaborative networks in health have been identified, including knowledge sharing, a positive social climate, and strong co-worker ties. In particular, knowledge sharing is important (2). Knowledge sharing strategies may include the distribution of knowledge, knowledge brokerage to encourage and support participation, and engagement of stakeholders in the network as well as knowledge governance to support the establishment of formal partnerships and policies (3).

Much of what is described in the manuscripts of this issue can be seen as the establishment, implementation, and exploitation of knowledge and data sharing. The featured collaborative networks in movement disorders can be seen as successful knowledge processing networks and should be conceptualized as such. In this sense, building of specialist networks is key in movement disorders or—the other way round—without disease networks, movement disorders will not move forward to develop new therapies and promote significant societal changes in support of the patients.

## Author Contributions

HG, AA, and LS are co-editors of the Networks in Movement Disorders topic and wrote the publication together. All authors contributed to the article and approved the submitted version.

## Funding

This work was generated with support from the European Reference Network for Rare Neurological Diseases—Project ID No 739510.

## Conflict of Interest

HG has received research support from the Deutsche Forschungsgemeinschaft (DFG), the Bundesministerium für Bildung und Forschung (BMBF), the Bundesministerium für Gesundheit (BMG), and the European Union (EU). The remaining authors declare that the research was conducted in the absence of any commercial or financial relationships that could be construed as a potential conflict of interest.

## Publisher's Note

All claims expressed in this article are solely those of the authors and do not necessarily represent those of their affiliated organizations, or those of the publisher, the editors and the reviewers. Any product that may be evaluated in this article, or claim that may be made by its manufacturer, is not guaranteed or endorsed by the publisher.
